# Impact of alloying iron pyrite by ruthenium on its band gap values and its insight to photovoltaic performance

**DOI:** 10.1016/j.heliyon.2023.e20270

**Published:** 2023-09-19

**Authors:** Eman A. Alghamdi, Refka Sai

**Affiliations:** a Department of Physics and Astronomy, King Saud University, Riyadh, 11451, Saudi Arabia; b Departement de Physique, Faculté des Sciences de Bizerte, Université de Carthage, Bizerte, 7200, Tunisia

**Keywords:** Bandgap, Optical properties, Iron pyrite, Spray pyrolysis, Photovoltaic cells

## Abstract

In pursuit of augmenting the band gap value of thin films composed of $FeS_{2}$ Pyrite, our study encompasses both theoretical and experimental investigations. Specifically, we sought to delve into the electronic and optical properties of $FeS_{2}$ alloyed with ruthenium, denoted as $Fe_{1-x}\;Ru_{x}\;S_{2}$, where x varied across a range of values (x = 0.3966, 0.1586, 0.0496, 0.0347, 0.0106, and 0.00). Our theoretical analysis employed the Linear Muffin-Tin Orbital technique within the Atomic-Sphere approximation (LMTO-ASA) framework, focusing on the density of states. In parallel, our experimental samples were fabricated via a cost-effective and straightforward method involving the sulfuration of amorphous iron oxide thin films, which were deposited through spray pyrolysis of an aqueous solution containing FeCl3.6H2O onto heated glass substrates at 400 °C. This comprehensive investigation sheds light on the influence of alloying on the atomic structure and the optical characteristics of $Ru_{x}Fe_{1-x}\;S_{2}$ samples. Utilizing X-ray diffraction (XRD) and optical characterizations, we observed a notable widening of the band gap of $FeS_{2}$, ranging from 0.90508 to 1.38 eV, when approximately 1.06% of the Fe atoms were replaced with ruthenium atoms (x = 0.0106 concentration of Ru). This finding holds significant implications for the potential applications of our samples in photovoltaic technologies.

## 1 Introduction

Amidst the ongoing discourse surrounding global warming, researchers and scientists are diligently striving to identify proficient sources of renewable energy. These sources must be adept at meeting the escalating demands of the market while concurrently aligning with environmentally conscientious practices. Among the viable options are biomass-derived energy, hydrothermal power, nuclear energy, and wind power. Additionally, solar energy stands out as an exceptional prospect, offering an efficacious, eco-friendly, and cost-effective alternative to the conventional, finite reservoirs of non-renewable energy. For that solar energy has gained a lot of recognition to be a good alternative to fossil fuels in terms of sustainability and reliability [[1], [2], [3], [4], [5]]. Many materials have found their applications in solar cell technology, but still popular material is silicon [6]. Other materials such as copper indium gallium selenide [7], cadmium telluride [8], perovskite materials [9], gallium arsenide [10] quantum dots [11] and different organic and polymer-based materials [12,13]. Wadia et al. conducted an extensive research study several years ago, examining a range of 23 potential materials for harnessing solar energy. Their findings unequivocally demonstrated that iron pyrite emerged as the most cost-effective option, surpassing all other materials in this regard [14,15]. Notably, it was affirmed that the cost associated with extracting silicon was a staggering 57 times higher than that of iron pyrite. Furthermore, Rahman et al. [15] subsequently underscored the cost-efficiency of iron pyrite in comparison to silicon, even when both materials were subjected to identical taxation and regulatory frameworks within the same country. In nowadays for these all-beneficial Iron Pyrite is an excellent material consider for solar cells that due its admissible band gap, its high absorption and its inexpensive cost. Its photovoltaic possibilities deepen its Earth abundance and nontoxicity. Iron pyrite is not only used in solar cells applications, it has interesting other applications in photodetectors, optoelectronic devices and optical storage. It is important, like other chalcogenide metals [16,17] has unique characterization with great electrical and optical properties to adapt it to any possible use. One attractive strategy to greatly lower the cost of photovoltaic energy conversion is to advance highly efficient photovoltaic cells using low-cost solution creation methods and naturally abundant materials. FeS_2_ pyrite is a potential candidate for renewable energy specifically in photovoltaic application due to its nontoxicity and high abundance [18].

FeS_2_ pyrite is essential one of metal sulfide minerals which is newly in the center of important experimental due of its low cost, its nontoxic constituent elements, its abundance, and its high absorption coefficient [19,20]. This attractive material has been the considerable.

Important of several scientists in the domain of solar cells since it is a favorable material of photovoltaic application [[21], [22], [23], [24], [25], [26], [27], [28], [29], [30], [31], [32], [33], [34]]. The results indicated that Solid State Schottky solar cells and pyrite photoelectrochemical cells (PEC) exhibit notable quantum efficiencies, reaching levels of up to 90%, accompanied by substantial short-circuit current densities ranging from 30 to 42 mA $cm^{-2}$, as reported in Refs. [19,20]. Notably, the semiconductor FeS2 pyrite possesses a band gap with a diminutive value of 0.95 eV, which, in the context of photovoltaic applications, is considered relatively small. This property merits attention in the realm of photovoltaic research [21]. For some reason, it is principal to present suitable methods to increase this band gap of FeS_2_ pyrite to progress pyrite-based photoelectrochemical solar cells and photovoltaic cells. The band gap of FeS_2_ pyrite able to be extend by adjoining cations or anions. Alloying by cation has the formula MxFe1-xS2 where M can be Zn, Ru et …, alloying by anion has the formula FeAxS2-x where A can be As, O etc. …. These alloying ways have been used highly [35,36]. Oxygen alloying FeS2 pyrite has been shown to enlarge its band gap [37]. Moreover, Sun in Ref. [35] affirmed that pyrite alloys with Zn or Os enlarges the band gap from 0.95 eV to 1.5 eV. Ruthenium (Ru) is one of cations able alloy iron pyrite and increases its band gap. Ru is an element that has been utilized widely for surface-coating of N-Chlorosuccinimide (NCS) and alloying because of its stability. The band energy of Ru-alloyed FeS_2_ pyrite, Ru_x_Fe_1-x_S_2_ can be enlarged from 0.95 eV to 1.8 eV because RuS_2_ pyrite has band gap about 1.8 eV [38]. Ceder and Sun [39] calculated the band gap of pyrite RuxFe1-xS2 and OsxFe1-xS2 utilizing density functional theory (DFT) calculation. They conducted an in-depth exploration into the potential for band gap engineering in pyrite ($\text{Fe}S_{2}$) through alloying it with various metallic elements, including but not limited to Zn, Os, Ru, Mg, Ba, among others, with the primary objective of enhancing its efficacy as a photovoltaic material. They found that the band gap of pyrite ${FeS}_{2}$ increases with add some percentage of Ru or Os. Also Jun Hu et al. [40] investigated by using DFT calculation, iron pyrite alloyed with oxygen; their obtained band gap shows an increase to 1.52 eV RuS2 pyrite has an indirect band gap [[41], [42], [43], [44]], while Iron pyrite has a direct band gap [[45], [46], [47], [48], [49]]. Also, some other new theoretical study done in Ref. [50]. In our recent work [51], we showed that Zinc alloyed iron pyrite able to enlarge band gap for small quantities of zinc. The band gap of $FeS_{2}$ pyrite increases to 1.15 eV by theoretical calculation and to 1.16 eV using experimental method by just adding 1% of Zinc.

The primary aim of this study is to elucidate the influence of integrated ruthenium (at both trace and elevated concentrations) on Iron Pyrite structural attributes. This investigation encompasses the augmentation of the band gap and other pertinent factors relevant to applications in the realm of solar energy. In the present manuscript, the feasibility of Ru alloyed iron pyrite was explored theoretically and experimentally in an essay to enlarge the band gap on the way to the optimal for an ameliorated photovoltaic application. We used spray pyrolysis for preparation of RuxFe1-xS2 samples. The formation of RuxFe1-xS2 was analyzed using XRD and Tauc Law to show the optical absorption diagrams. We utilized the Linear Muffin-Tin Orbital Method in the Atomic-Sphere approximation LMTO-ASA calculation to investigate the impact of different percentage of Ru (x = 0.3966, 0.1586, 0.0496, 0.0347, 0.0106 and 0.00) on band gap, optical and electronic structure properties.

Given the significance of semiconductors within the domain of solar energy, the concluding segment of this paper entails the practical application of our findings in this context.

## 2 Experiment

We utilized spray pyrolysis technical for formation of our samples, which this method is well described in Refs. [52,53]. In our strategy, we use of a mixture resulted from the dissolution of 8.5 g of $RuCl_{3}.\;3H_{2}O$ and $FeCl_{3}.6H_{2}O$ within a molar ratio as $FeCl_{3}.6H_{2}O$: $RuCl_{3}.\;3H_{2}O$ = x:1 − x (x = 0.3966, 0.1586, 0.0496, 0.0347, 0.0106 and 0.00) through 7 min substrates for temperature of heated at 400 °C. The transport rate is about 50 cm and 7 mL/min. We obtained in the aqueous solution the following reactions:

$$FeCl_{3}.6H_{2}O\to \;{Fe}^{3+}+3Cl^{-}+6H_{2}O$$

$$RuCl_{3}.3H_{2}O\to \;{Ru}^{3+}+3Cl^{-}+3H_{2}O$$

**Fig. 1:**
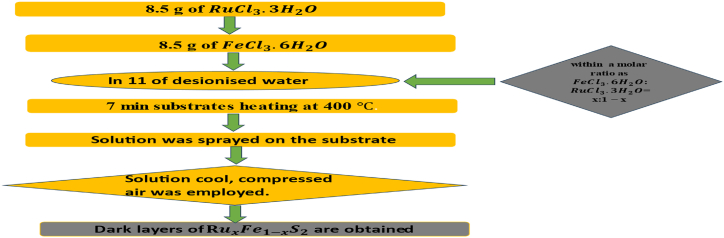
Growth of monocrystal of $Ru_{x}{\text{Fe}}_{1-x}S_{2}$ by spray pyrolysis.

Wich can give us $Ru_{x}{\text{Fe}}_{1-x}S_{2}$ phases in our ${FeS}_{2}$ pyrite layers. Compressed air was employed as the carrier gas. Dark layers are obtained. Fig. 1 presents the procedure of treatment of samples by spray pyrolysis. We succeed to obtain $Ru_{x}{\text{Fe}}_{1-x}S_{2}$ layers for different percentage of ruthenium for x = 0.3966, 0.1586, 0.0496, 0.0347, 0.0106 and 0.00.

## 3 Results and discussions

### 3.1 XRD characterization

**Fig. 2:**
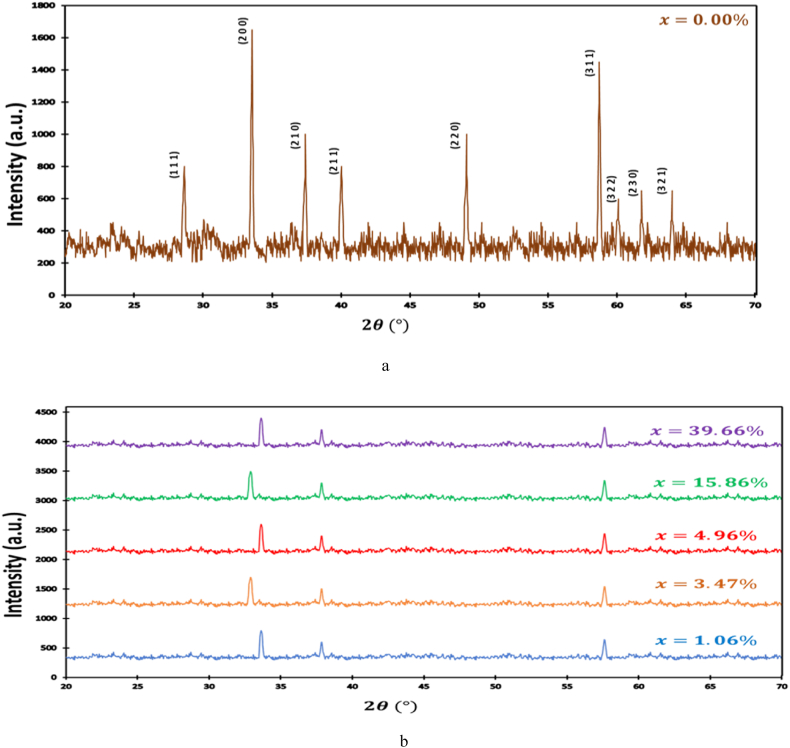
a: XRD diagram of $FeS_{2}$ pyrite Fig. 2bXRD patterns of $Ru_{x}Fe_{1-x}S_{2}$.

To study the possible modification in crystallinity induced by the alloy with ruthenium, we conducted an XRD analyze. The structures of $Ru_{x}{Fe}_{1-x}S_{2}$ *a*re examined by powder X-ray diffraction (XRD) utilizing Siemens D500 diffractometer (CuK α radiation λ = 1.54201 Å). Parameters lattice and the crystal structural are obtained utilizing the Rietveld way were utilized PDXL program. The XRD diagrams are shown in Fig. 2a,b.Fig. 2a correspond $\text{Fe}S_{2}$ pyrite (x = 0.00), typical diffraction peaks corresponding to plan (111), (200), (210), (211), (220), (311), (222), (230) and (321); and well match to the predictable diffraction details of the $\text{Fe}S_{2}$ pyrite (JCPDS card n °: 035–0077; space group: Pa3). Fig. 2b representative diffraction peaks correspond to the (200), (210), and (311) planes. The resulting structures of $Ru_{x}{\text{Fe}}_{1-x}S_{2}$ are a cubic crystal structure be in the space group Pa $\bar{3}$ (205) for different percentage of Ru.

The X-ray diffraction (XRD) analysis reveals notable alterations in the diffraction patterns of $Ru_{x}\;Fe_{1-x}\;S_{2}$ as the Ru content increases. Specifically, a shift towards both larger and smaller diffraction angles is observed, accompanied by a slight broadening of the peaks. This broadening phenomenon can be ascribed to the reduction in nanocrystal size with the increasing proportion of Ru. This observation substantiates our hypothesis that the introduction of Ru into $FeS_{2}$ pyrite leads to an expansion of the lattice constant, owing to the fact that $RuS_{2}$ possesses a larger lattice constant compared to Iron pyrite. Importantly, the XRD patterns exhibit no indications of impurities such as marcasite, pyrrhotite, or greigite, affirming the high purity of the synthesized materials.

Furthermore, the crystal structure of Ru-alloyed iron sulfide, denoted as $Ru_{x}\;Fe_{1-x}\;S_{2}$, conforms to the pyrite phase within a cubic lattice. The resultant parameters, including atom sulfur positions (u) and lattice constant (a), are summarized in Table 1 for reference.

**Table 1:** Parameters Cells determined by using PDXL Program.

x	0.3966	0.1586	0.0496	0.0347	0.0106	0.00
Cubiclattice a(Å)	5.429	5.427	5.423	5.421	5.419	5.417
υ(positionparameter)	0.111	0.111	0.112	0.112	0.113	0.114
u(Sulfur position)	0.389	0.389	0.388	0.388	0.387	0.386

The XRD patterns show the impact of doping Iron pyrite By ruthenium in crystallites size, in our case crystallites size increase with increases of ruthenium percentage and that much with work done in Refs. [[54], [55], [56]]. According to the procedure of fabrication, we can have said that our samples are with a good crystallinity and an enlarge of the band gap value was found after substituting with Ruthenium.

### 3.2 Optical properties

**Fig. 3:**
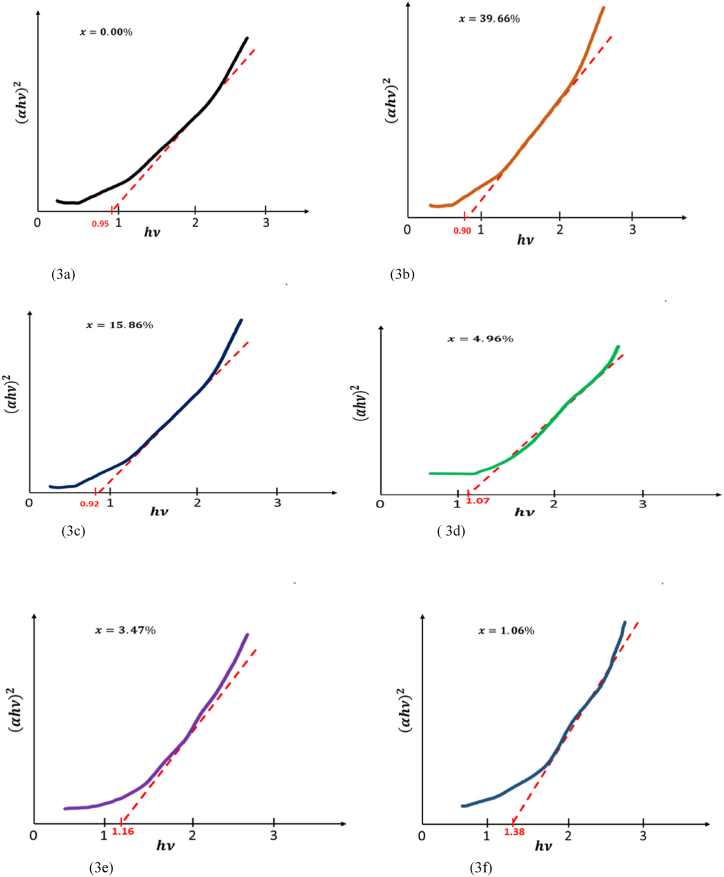
(a, b, c, d, e and f): Plots of ${\left(\alpha h\nu \right)}^{2}$ versus hν..

The plots of ${\left(\alpha h\upsilon \right)}^{2}$ versus the photon energy hυ were obtained by using SHIMADZU 3100s spectrophotometer. Our figures are presented in Fig. 3(a, b, c, d, e and f) our diagrams allow straight line, indicate that Ru-alloyed FeS_2_ sample has a direct band gap energy. We can observe that band gap increases only for tiny percentages for x = 0.0496, 0.0347 and 0.0106 increases to 1.07 eV,1.16 eV and 1.38 eV. Through our experimental, we succeed o enlarge band gap of iron pyrite to 1.38 eV for smallest percentage of ruthenium (∼ 1.06%). Band gap values are listed in Table 2.

**Table 2:** band gap values of Ru-$FeS_{2}$ versus the added amount of Ruthenium.

x	0.3966	0.1586	0.0496	0.0347	0.0106	0.00
Bandgap Eg(eV)	0.90	0.92	1.07	1.16	1.38	0.95

**Fig. 4:**
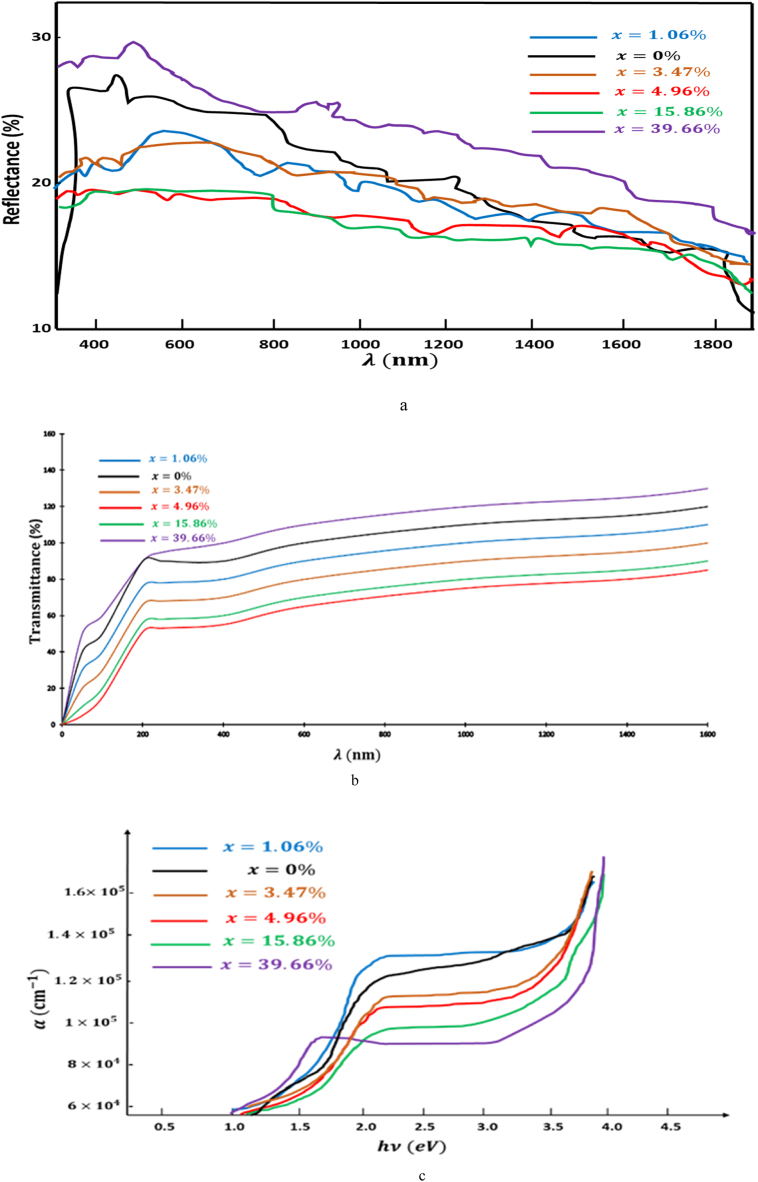
a: Reflectivity spectra Fig. 4bTransmittance spectra Fig. 4cAbsorption coefficients.

Fig. 4 depicts a graphical representation illustrating the reflectivity and transmission coefficients of the samples in their as-prepared state, covering a spectral range spanning from 240 nm to 1800 nm. It is noteworthy that the heat treatment induced a minor reduction in reflectivity, as depicted in Fig. 4a, while Fig. 4b reveals a notable decrease in transmittance as a result of this treatment. The absorption coefficient is high ($\alpha >1.2.10^{5}{cm}^{-1}$) (Fig. 4c).

**Fig. 5:**
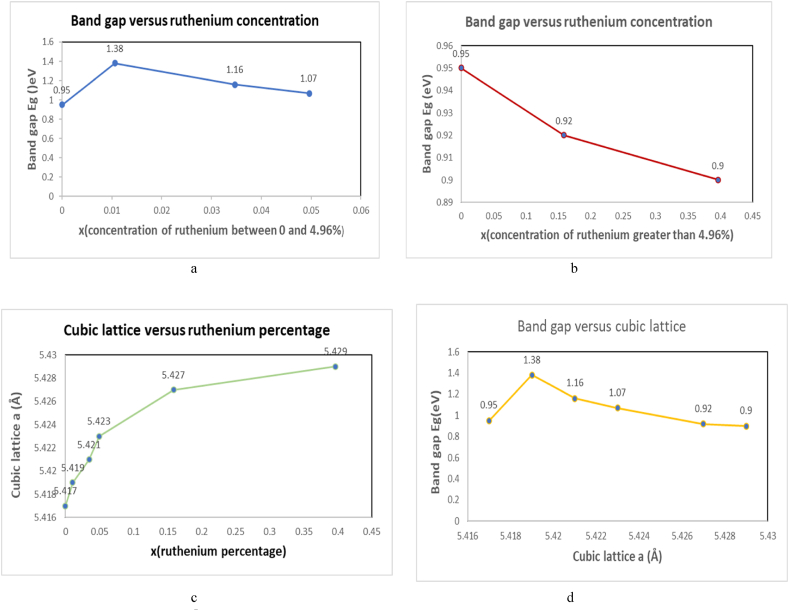
a: Band gap versus Ru concentration Fig. 5b: Band gap versus Ru concentration Fig. 5cCubic lattice versus ruthenium concentration Fig. 5d: band gap versus cubic lattice.

According to the procedure of fabrication our results show performant value the band gap which was estimated of about 1.38 eV for 1.6% of amount of ruthenium. This can be accepted as an optimum value for the photovoltaic solar cells application. Our results are listed in Fig. 5(a, b, c and d), which We notice that the band gap decreases with increases of constant lattice (Fig. 5d), which cubic lattice a increases with increases of ruthenium percentage (Fig. 5c). Also, we have for ruthenium percentage greater than of 4.96% the band gap decreases, it ranges between 0.90 and 0.92 eV in (Fig. 5b). The most important results are that we show enlarge band gap of iron pyrite is only by small concentration of ruthenium for x = 0.0106, 0.0347 and 0.0496 (Fig. 5a). We succeeded in increasing the band gap of Iron pyrite to 1.07 eV–1.16 eV and 1.38 eV by alloying with small concentration of ruthenium.

### 3.3 DFT calculation

In our computational approach, we employed Density-Functional Theory [57,58] with a specific focus on the linear muffin-tin orbital method within the atomic-sphere approximation (LMTO-ASA). A comprehensive elucidation of the LMTO-ASA techniques has been extensively documented in various scholarly reports [[59], [60], [61]]. This study adheres to the principles of self-consistent band calculations, as they represent the foundational aspects of density functional theory [57], operating within the local density approximation [62]. We incorporated numerical methodologies to facilitate the treatment of electron-ion interactions within the pseudopotential framework [63]. Furthermore, the Hamiltonian within the Atomic Spheres Approximation is uniquely determined by the potential variable, which subsequently yields moments derived from the Hamiltonian's eigenvectors. Notably, this approach simplifies the potential (denoted as P) [64,65].

Our computational process initiated with the determination of the potential variable for all atomic spheres, with $V_{MTZ}$ representing the muffin-tin potential constant at the centers of the atomic spheres for Fe, Ru, and S. The specific values of $V_{MTZ}$ are documented in Table 3. It is noteworthy that the primary sphere packing attained an initial coverage of 80.1%, which was subsequently scaled up to 94%. This adjustment in sphere packing serves the dual purpose of minimizing the total number of iterations in the computational system and reducing overlap among the spheres centered on Ru, Fe, and S.

**Table 3:** Specific values of VMTZv.

x	0.3966	0.1586	0.0496	0.0347	0.0106	0.00
$V_{MTZ}$	−0.679772	−0.715219	−0.637329	−0.698774	−0.737464	−0.737209

**Fig. 6:**
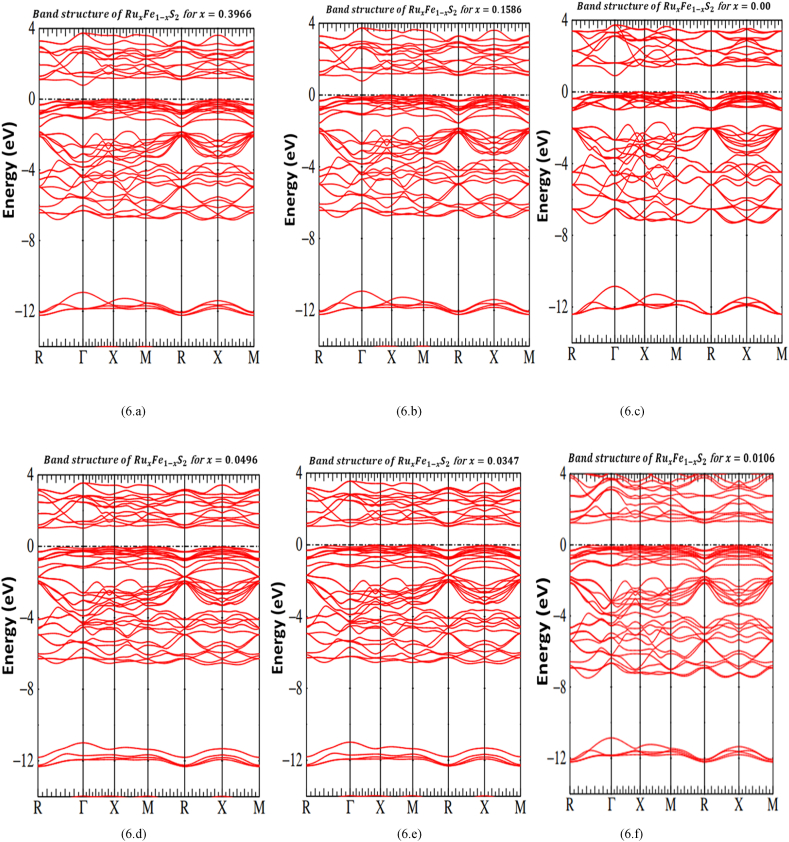
(a, b, c, d, e and f): Band structure of $Ru_{x}Fe_{1-x}S_{2}$ for different x.

Fig. 6(a, b, c, d, e and f) show energy band diagram of $Ru_{x}{Fe}_{1-x}S_{2}$ for different added amount of Ruthenium. All figures present a full diagram of twice valence bands and conduction bands in an energy range between −14 eV and +4 eV. We divide this band structure in order of increasing energy. The number of electrons at occupied bands are 80 valence electrons. We notice bands related with antibonding and bonding of orbitals on $S_{2}$ pairs. The bands have S3s character, it has a maximum energy of −11 eV, which S3s characters are predominant. The bands compose of sulfur 3p levels get going estimably at −7 eV, their principal bands comprise an additional character due to the levels of Fe 3d and a tiny admixture of the levels of Ru 4d. This bands compose within bonding S 3p and Ru $e_{g}$ and Fe $e_{g}$. The Ru 4d $t_{2g}$ and Fe 3d $t_{2g}$ bands are just under the Fermi level. The conduction bands are split beyond the Fermi level, these bands have S 3p and Ru 4d $e_{g}$ and Fe 3d $e_{g}$ characters.

Our calculations indicate that of $Ru_{x}{Fe}_{1-x}S_{2}$ for different added amount of Ruthenium is a direct gap semiconductor. The values of band gap are presented in Table 4. Fig. 3 (a, b, c, d, e and f) show that the optical measurement of $Ru_{x}{Fe}_{1-x}S_{2}$ for different added amount of Ruthenium have a direct gap of 0.90 eV, 0.92 eV, 1.07 eV, 1.16 eV 1.38 eV and 0.95 eV whichever is in great agreement within our theoretical calculations band gap 0.84 eV, 0.87 eV, 1.00 eV, 1.05 eV, 1.35 eV and 0.90 eV.

**Table 4:** Ru-$FeS_{2}$ band gap calculated values.

x	0.3966	0.1586	0.0496	0.0347	0.0106	0.00
Bandgapcalculated(eV)	0.84	0.87	1.00	1.05	1.35	0.90

Notice that our calculation employs the remarkable increase of band gap only for tiny percentages of ruthenium especially for the smallest amount (x = 0.0106) which the gap enlarges from 0.90 eV to 1.35eV. we show that an increase of band gap calculated only for small percentages of amount of ruthenium (1.06%, 3.47% and 4.96%) band gap about 1.35 eV, 1.05 eV and 1 eV. Band gap calculated decreases when the amount of ruthenium increases, which will be 0.84 eV and 0.87 eV. A remarkable change of band gap calculated depend constant lattice change, band gap value decreases with increases of constant lattice. We conclude that the use of Ru as an alloying for iron pyrite is the perfect way as previewed by many researchers [[66], [67], [68]].

We conclude that the presence of low concentration of ruthenium (1.06 %, 3.4 % 7 and 4.96% 0f Ru) alloy will shift the band gap up by ∼ 0.8 eV, ∼0.6eVand∼ 0.5 eV; at the same time, it does not change the state of Iron pyrite. Also, we conclude that impact of alloy in crystalline structure which cell parameter changes for each percentage, we mark that only for small percentage of Ru we get cell parameter close to cell parameter of Iron pyrite.

### 3.4 Photovoltaic application: Modeling the ITO/ZnO/ $Ru_{x}{Fe}_{1-x}S_{2}$/MoO3/Au/Ag devices

**Fig. 7:**
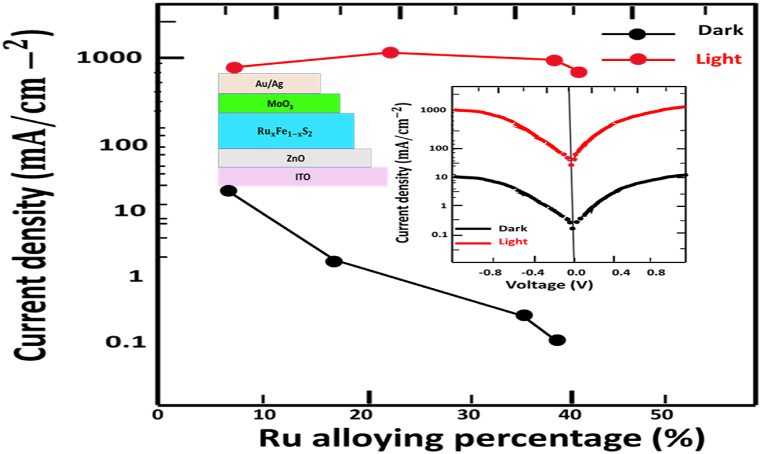
Variation of current density of $Ru_{x}Fe_{1-x}S_{2}$ as a function of percentage of Ru..

It is important to look in the impact of alloying iron pyrite in photovoltaic. IPOs solar cells or inorganic photovoltaic are the most commercially successful and are also widely utilized at solar cells systems. The synthesized $Ru_{x}{Fe}_{1-x}S_{2}$ films were estimated for the utilization in solar cell. We matched an ITO/ZnO/ $Ru_{x}{Fe}_{1-x}S_{2}$/MoO3/Au/Ag to investigate the improvement in photovoltaic devices properties realize by increases of band gap for all different percentages added of Ru. The structure form is shown in Fig. 7. All substrates are cleansed in isopropanol, soap and water for 15 min. Recently ZnO has being the main candidate as organic photovoltaic cell [43] because its ability stability and improvement. In addition, a MoO3 thin film was employed to serve as an effective electron-blocking layer or hole-transporting material, with the primary objective of mitigating electron-hole recombination, as elucidated in Ref. [44]. The schematic diagram illustrating the experimental characterization involved applying a voltage within the range of −1 to 1 V while measuring the dark current in a structured model comprising ITO/ZnO/ $Ru_{x}Fe_{1-x}S_{2}$/MoO3/Au/Ag, as illustrated in Fig. 7. A remarkable observation in our current density diagram is a particular dark current reduces with enlarging percentage of Ruthenium added. The dark current reduces from 60.2 to 3.0 mA cm-2 with enlarging ruthenium percentage as shown in Fig. 7. Our outcome agrees with the supposition that an enlarged band gap energy of the semiconductor offers lower dark current. With the slightly enlarged photocurrent and dramatically lower current, the alloy of the ruthenium into $FeS_{2}$ pyrite derived an interesting photodetector application. Moreover, incorporation of Ru into Iron Pyrite is important for photovoltaic application. Notice that fabricates $Ru_{x}{\text{Fe}}_{1-x}S_{2}$ improved efficiency can be expected for multispectral photovoltaic cells.

## 4 Conclusion

Our experimental and our calculations demonstrated that alloying Iron pyrite with small concentration (1.06 %, 3.4 % 7 and 4.96%) of ruthenium is an effective method to enlarge band gap of Iron pyrite. A low-cost process was select to fabricate $Ru_{x}{Fe}_{1-x}S_{2}$ films containing great properties for photovoltaic devices. An improvement of our fabricate $Ru_{x}{Fe}_{1-x}S_{2}$ films by spray pyrolysis in increase the band gap of Iron pyrite to 1.38 eV,1.16 eV and 1.07eV. The incorporation of ruthenium conserves the states of band gap (direct band gap), electronic and optical properties. Our experimental and our calculation show that only small percentages of Ru able to increase the band gap especially smallest concentration x = 0.0106, $Ru_{.0.0106}{Fe}_{0.9894}S_{2}$ has a band gap optical ∼ 1.38 eV and 1.35 eV band gap calculated which present a good value for solar cell application. Also, our study shows the dependence between of constant lattice and band gap, witch we can have said that band gap decreases with increases of constant lattice. Only for lattice constant about 5.419 Å , 5.421 Å and 5.423 Å band gap of Iron pyrite increases. Our work shows the effect of parameter cells on band gap and photovoltaic performance. An increase of photoresponse with Ru alloying was shown in the $Ru_{x}{Fe}_{1-x}S_{2}$ devices resulting from the lower dark current.

In summary, our study has substantiated the substantial expansion of the band gap of iron pyrite through the judicious incorporation of trace quantities of ruthenium, ranging between 1% and 5%. Notably, the novelty of our findings lies in the synergistic application of experimental techniques and theoretical analysis, which has yielded comprehensive and robust outcomes.

## Data availability statement

Data will be made available on request.

## Author contribution statement

Eman A Alghamdi; Refka Sai: Conceived and designed the experiments; Performed the experiments; Analyzed and interpreted the data; Contributed reagents, materials, analysis tools or data; Wrote the paper.

## Declaration of competing interest

The authors declare that they have no known competing financial interests or personal relationships that could have appeared to influence the work reported in this paper.
